# Current progress of functional nanobiosensors for potential tuberculosis diagnosis: The novel way for TB control?

**DOI:** 10.3389/fbioe.2022.1036678

**Published:** 2022-12-15

**Authors:** Xuran Yang, Shuhao Fan, Yuhe Ma, Hui Chen, Jun-Fa Xu, Jiang Pi, Wandang Wang, Guanghui Chen

**Affiliations:** ^1^ Department of Clinical Medicine Laboratory, Affiliated Xiaolan Hospital, Southern Medical University, Zhongshan, China; ^2^ Guangdong Provincial Key Laboratory of Medical Molecular Diagnostics, The First Dongguan Affiliated Hospital, Guangdong Medical University, Dongguan, China; ^3^ Institute of Laboratory Medicine, School of Medical Technology, Guangdong Medical University, Dongguan, China

**Keywords:** nanomaterials, nanobiosensor, tuberculosis, diagnosis, recent progress

## Abstract

Tuberculosis (TB), induced by the foxy *Mycobacterium tuberculosis* (Mtb), is still one of the top killers worldwide among infectious diseases. Although several antibiotics have been developed to significantly relieve the tuberculosis epidemics worldwide, there are still several important scientific challenges for tuberculosis. As one of the most critical issues for tuberculosis control, the accurate and timely diagnosis of tuberculosis is critical for the following therapy of tuberculosis and thus responsible for the effective control of drug-resistant tuberculosis. Current tuberculosis diagnostic methods in clinic are still facing the difficulties that they can’t provide the rapid diagnostic results with high sensitivity and accuracy, which therefore requires the development of more effective novel diagnostic strategies. In recent decades, nanomaterials have been proved to show promising potentials for novel nanobiosensor construction based on their outstanding physical, chemical and biological properties. Taking these promising advantages, nanomaterial-based biosensors show the potential to allow the rapid, sensitive and accurate tuberculosis diagnosis. Here, aiming to increase the development of more effective tuberculosis diagnostic strategy, we summarized the current progress of nanobiosensors for potential tuberculosis diagnosis application. We discussed the different kind diagnostic targets for tuberculosis diagnosis based on nanobiosensors, ranging from the detection of bacterial components from *M. tuberculosis*, such as DNA and proteins, to the host immunological responses, such as specific cytokine production, and to the direct whole cell detection of *M. tuberculosis*. We believe that this review would enhance our understandings of nanobiosensors for potential tuberculosis diagnosis, and further promote the future research on nanobiosensor-based tuberculosis diagnosis to benefit the more effective control of tuberculosis epidemic.

## Introduction

Tuberculosis (TB), caused by the foxy *Mycobacterium tuberculosis* (Mtb), remains to be one of the most catastrophic diseases since human history. According to the global TB report in 2021, the newly diagnosed cases of TB were up to 10 million with an estimated 1.4 million deaths in 2020 (World Health, 2021). Although numerous advances have been made in understanding of the epidemiology, risk factors, pathophysiology, diagnosis and treatment, TB is still one of the top 10 causes of death among diseases worldwide, ranking above HIV/AIDS. Although several antibiotics have been developed to significantly relieve the TB epidemics, there are still several important scientific issues for TB. As one of the most critical issues for the control of TB and drug-resistant TB epidemics, the diagnosis of TB is critical for the timely therapy of TB.

Currently, there are several conventional methods that are widely used for *Mtb* detection or TB diagnosis, which have made plenty of contributions in the control of TB epidemic. However, these current TB diagnostic methods are still facing some difficulties that they can’t provide the rapid diagnostic results with high sensitivity and accuracy simultaneously. Ziehl-Neelsen (ZN) smear microscopy is the most common used method for clinical TB diagnosis, which is inexpensive with low bio-safety standards, but it shows restricted sensitivity when the bacterial load of Mtb is lower than 10,000 organisms/ml in sputum samples (Steingart et al., 2006; Desikan, 2013). Mycobacterial cultures is a very accurate TB diagnotic strategy that offers improved sensitivity up to 60%, but it is time consuming and needs a bio-safety level-3 laboratory theoretically, which introduces severe constrains on the prompt TB diagnosis (Dolin et al., 1994; Smith, 2003; Onyango, 2011; Wang et al., 2014; World Health, 2015a; World Health, 2015b). The reactive tuberculin skin testing (TST) and interferon gamma release assays (IGRAs) provide methods for the detection of latent TB (LTB) through targeting memory T-cell receptor previously-sensitized by prior TB infection. TST is inexpensive and widely used, but it requires 48–72 h to obtain the results based on two patient visits and the results are very easily to be disturbed by some non-Mycobacterial (NTM). IGRAs based detection method possesses high sensitivity and specificity, but it might introduce false positive diagnosis in subjects with high concentration of IFN-γ. The high cost and the requirement of special laboratory settings and well-trained staffs also partially restrict the application of IGRAs. The Xpert Mtb/RIF assay, a new rapid diagnostic test based on nucleic acid amplification, works very well in diagnostic of *Mtb* and rifampicin resistance in 2 h (El-Samadony et al., 2017), but it is not only restricted by its relatively low sensitivity and high false positivity rates in same specific conditions (Arend and van Soolingen, 2018), but also show limitations for the diagnosis and management of polyresistant pulmonary tuberculosis (Hopmeier et al., 2020).

In recent decades, nanomaterials are widely used in biomedical fields and have been developed for tissue regeneration (Xue et al., 2022), anti-infection (Alotaibi et al., 2022) (Lin et al., 2022), antitumor (Wang et al., 2022). Based on their excellent physical, chemical and biological properties, nanomaterials have also been proved to show great potential in the development of new detection technologies. For example, Guthula et al. (2022) designed a method for the determination of fiber-optic gold nanolinked adsorbents based on PCR-free DNA from human gastric tumor tissues and cell lines, which has shown good sensitivity, accuracy and short detection, and can be used as an alternative to PCR with great potential for diagnostic biosensor fabrication.

In addition to the relevant detection of viral and cancer factors, more and more nanomaterials with attractive functions have been developed as promising tools for sensitive, rapid and accurate detection for TB related-samples as the unique properties of nanomaterials. Such manipulations enable nanomaterials to operate specific analytical functions and hence their applications as biosensors (Rai et al., 2016), which might benefit the development of novel TB diagnostic strategies. In this review, we systemically summarized the current progress of nanobiosensors for potential TB diagnosis application. Firstly, we introduced the basic principles of how nanobiosensor works for potential diagnosis. In the second part, we seperately discussed the current progress of nanobiosensors targeting DNA and protein of Mtb for TB diagnosis. Thirdly, we also introduced the development of nanobiosensors for Mtb infection associated cytokins production, followed the current progress of nanobiosensors for whole cell of Mtb analysis. At last, we discussed the perspectives of the functional nanobiosensors for potential tuberculosis diagnosis, which might promote the following research on nanobiosensor-based TB diagnosis and further benefit the development of diagnostic strategy for more effective control of TB epidemic.

### How nanobiosensor works for potential diagnosis

The landscape of TB diagnostic strategy is still restricted by the lack of ideal strategy with low cost, high sensitivity, accurate and rapid diagnosis. The development of nanotechnology allows the possible development of nanobiosensors, which allow the detection of biological molecules or components. A nanomaterial based biosensor is actually a kind of integrated device that basically consists of a biological recognition element, transducer, and processor, which can specifically recognize the target bio-analytes we prefer to be detected.

**FIGURE 1:**
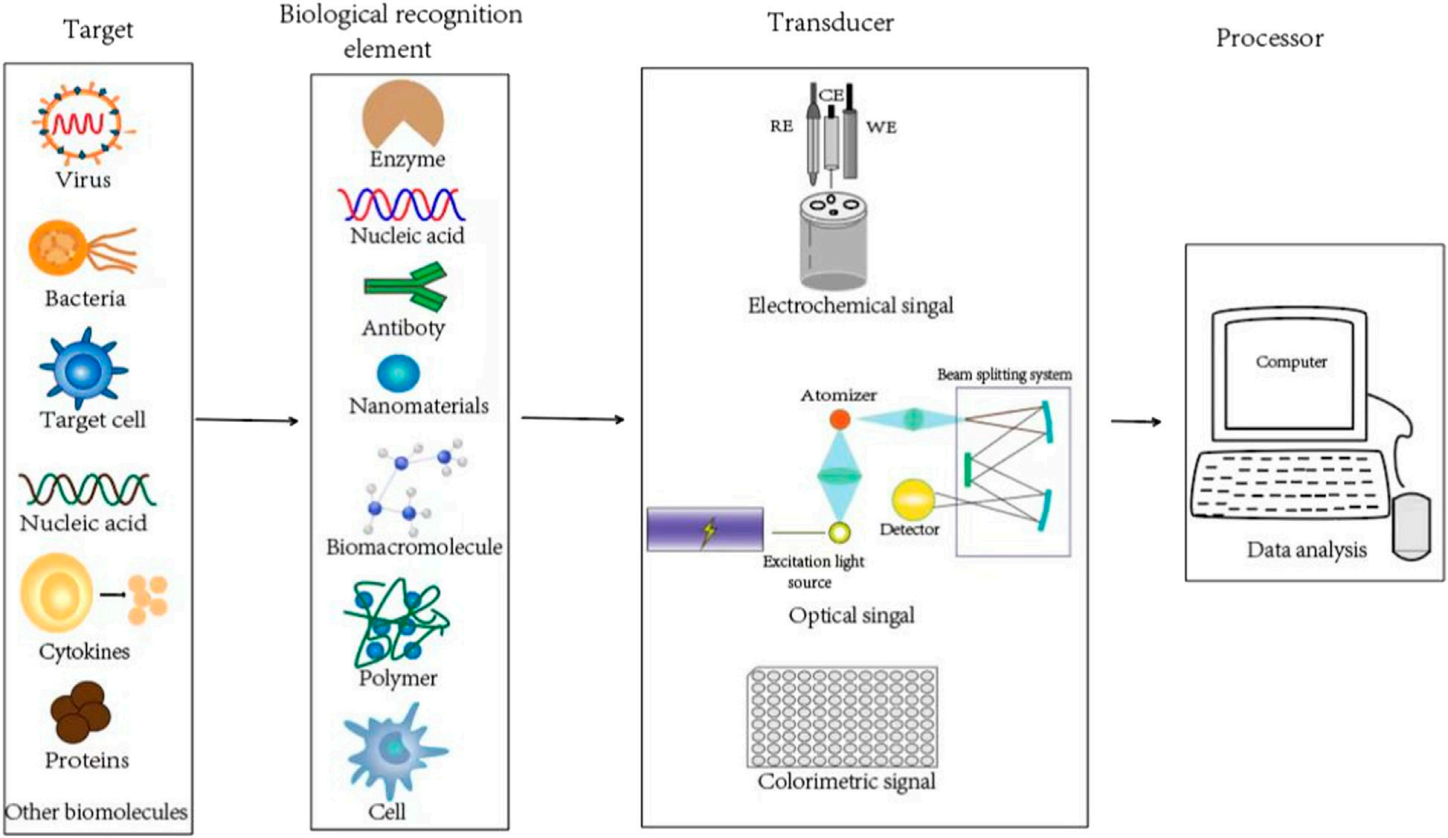
Schematics of the biological recognition element, transducer, and processor for typical nanobiosensors to detect the target bio-analytes.

As shown in Figure 1, the target bio-analytes include nucleic acids (DNA, RNA), proteins such as enzymes, antibodies and antigens, immune cell cytokines, and whole cells (bacterial or virus), which can be captured by complementary bio-recognition probes. The typical biological recognition element for nanobiosensors is always formed by some nanomaterials, biomacromolecules (such as nucleic acid and proteins), polymers and even cells, which can be applied to specifically recognize the target bio-analytes. By establishing the linkage between specific and efficient bio-interaction with corresponding signals (such as electrical, fluorometric, luminometric or colorimetric signals), the levels or activities of elements involved in the biological recognition can be converted into specific signals by the transducers, which can be further amplified, quantified, displayed and analyzed (Abu-Salah et al., 2015; Gupta and Kakkar, 2018). Depending on the different detection parameter and theory, transducers could be classified into different strategies, such as optical (like SPR), piezoelectric (such as quartz crystal microbalance), electrochemical, thermometric, magnetic or micromechanical signal (Gupta and Kakkar, 2018). At last, the processor could translate the transducer signals into qualitative or quantitative data that could reflect the levels or activities of the targeted bio-analytes.

**FIGURE 2:**
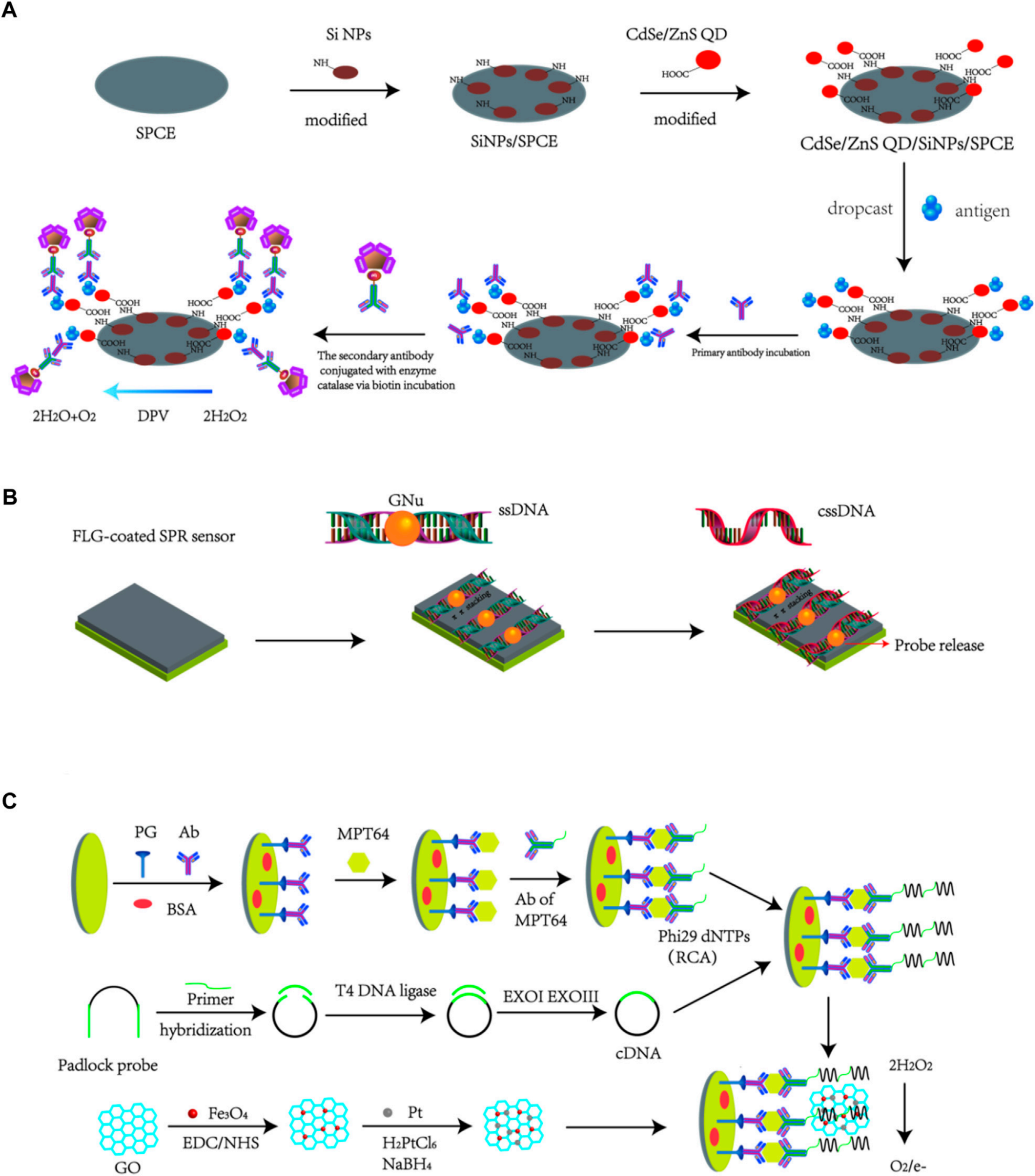
Basic approaches to design nanobiosensors: **(A)** Modification of nanoparticles onto electrode surfaces to construct functional electrode-based biosensors (Mohd Bakhori et al., 2019). **(B)** Design of nanosystems to construct nanoprobes that can be used for nanobiosensor application (Prabowo et al., 2021). **(C)** Hybrid method to construct Nanobiosensors using both approaches **(A,B)** (Gou et al., 2018).

Taking the advantages of various unique properties like large loading volume, better electrical communication ability, stability and heat conductivity, combining nanomaterials with transducers could significantly enhance the diagnostics sensitivity, which makes nano-biosensing detection method a promising strategy for TB diagnosis. Up to now, two widely known approaches are always applied to design nanobiosensors: i.e., the modification of electrode surfaces by nanomaterials (Approach 1, example is presented as Figure 2A), or the fabrication of signal nanoprobes by nanomaterials to enhance generated signal (Approach 2, example is presented as Figure 2B). There are also some nanobiosensors constructed using both amplification approaches (hybrid biosensing, i.e., Approach 3, example is presented as Figure 2C). We also introduced the examples for the specific principles for these basic approaches as shown as in Figure 2. Nano-biosensing diagnostics about TB detection contain different kinds of bio-analytes, such as DNA, protein, cytokines and even whole bacteria. In this review, the most recent research advances in the field of nano-based biosensors developed for TB detection were summarized.

### Nanobiosensors targeting DNA of Mtb for TB diagnosis

In recent decades, the methods for DNA detection has attracted increasing attentions for early diagnosis of diseases. The DNA contents of Mtb can be detected by some techniques, and some most widely used techniques are polymerase chain reaction (Sanjuan-Jimenez et al., 2013; Vinuesa et al., 2018), microarray assay (Fukushima et al., 2003) and cycling probe technique (Pahwa et al., 2005) in the research laboratory. However, the existing methods were still not widely used in clinical laboratory diagnosis due to some limitations, such as complexity, time-consuming analysis duration and high cost. The rapid development of nanomaterials allows researchers a new way to design more effective detection methods for TB infection.

As mentioned before, researchers usually develop nano-biosensors detection methods for TB detection based on the Approach 1–3 alone or together. Prabowo et al. (2021) introduced two distinct gold nanoparticle (AuNPs) structures, including gold nano-urchins (AuNu) and gold nanorods (AuNr), to bind the specifically designed single-stranded DNA probe (ssDNA) against the complex DNA of Mtb. These two kinds of AuNPs-ssDNA probes were adsorbed onto the few-layer graphene (FLG) through the π-π stacking force for SPR sensor coating, which allowed the hybridization interactions with their complementary single-stranded DNA (cssDNA). During the hybridization interactions, the strong hydrogen bond force between cssDNA and ssDNA could gradually remove the AuNPs-ssDNA from the graphene surface, which could result in a negative ΔSPR signal that could be detected by the SPR sensor. This method shows an estimated limit of detection (LOD) of ∼24.5 fM cssDNA for GNus SPR signal (sensitivity), and a lower LOD of 8.2 pM cssDNA for GNrs SPR signal (sensitivity). The overall integrated approach of the graphene-based SPR sensor and AuNPs-assisted DNA detection method provided the proof-of-concept results for the further development of potential TB screening strategy. The DNA hybridization study based on the SPR sensor indicated that rough and spikey GNu could significantly reinforce the DNA hybridization signal than the smooth GNr. The DNA hybridization detection strategy was assisted by GNu to reach a outstanding limit of detection (LoD) at femtomolar level. But it is worth to note that the obtained results were all based on pure DNA samples, without any evidences to prove its applicability for the complicated clinical sample analysis. More works are still needed to test the ability of this nanobiosensor for samples isolated from patients.

To increase the sensitivity of TB diagnostic method, Hatami et al. (2020) firstly decorated the glassy carbon electrode (GCE) using zinc oxide (ZnO) before AuNPs deposition. The combination of ZnO with AuNPs could increase the surface area of the obtained electrode for better electrical communication compared to GCE, which bring higher sensitivity for DNA analysis. Later, the platform was modified with thiolated probe DNA by covalently conjugation, and then, the GCE/ZnO/AuNPs/ssDNA nanobiosensor was used to capture the targeted TB DNA. After incubated with sample solution for 45 min, the DNA nanobiosensor was analyzed by electrochemical impedance spectroscopy (EIS) with a LOD of 1.8 pM. Compared with an equimolar concentration for completely matched DNA (target), the corresponding ΔI_p_ values for double-base mismatched, three-base mismatched and non-complementary strands were only 48.8%, 27.0%, and 4.93%, indicating the high recognition ability of the proposed nanobiosensor towards TB DNA. In addition, the DNA nanobiosensor showed high stability as the measuredΔI_p_ decreased only 4.25% after storing at 4°C for 10 days. This work proposed label-free DNA nanobiosensor displayed high selectivity and sensitivity with wide linear range and picomolar LOD for TB DNA analysis. However, this work just tested the performance of the nanobiosensor for pure TB DNA sample analysis, no further attempts to detect the more complicated clinical samples.

Bai et al. (2019) also used the GCE electrode decorated with a biotin-avidin system that immobilizing with abundant capture probes (CPs) as platform to detect TB DNA. The AuNPs were immobilized into a fullerene nanoparticles and nitrogen-doped graphene nanosheet (G-nano-C_60_/NGS), which was subsequently labeled by signal probes (SPs) for signal amplification. Later, a typical sandwich hybridization was produced in the presence of target DNA of Mtb, which was further incubated with tetraoctylammonium bromide (TOAB) to get discriminating responses. This electrochemical DNA-based nanobiosensor showed a broad linear range for the determination of Mtb DNA at a range of 10 fM-10 nM with a low LOD of 3 fM. Additionally, this prepared nanobiosensor can not only distinguish mismatched DNA sequence, but can also differentiate Mtb from other pathogens like *Staphylococcus aureus* and *Candida albicans*, indicating its strong Mtb DNA selectivity and specificity. Additionally, SPs labeled G-nanoC_60_ showed a weak peak compared to SPs labeled G-nano-C_60_/NGS, indicating that this G-nano-C_60_/NGS nanosystem could enhance the sensitivity of the electrochemical biosensor probably due to the presence of NGS to provide larger surface and facilitate electron transfer. Moreover, the steps for biosensor preparing was simplified as this nanohybrid was directly used to generate and enhance signals. With these merits, this electrochemical biosensor for DNA analysis is able to detect IS6110 fragments with fmol sensitivity, high specificity, reproducibility and stability, which therefore might be of great use in clinical practice. However, more works that directly analyzing the clinical samples should be further conducted to expand this nanobiosensor for clinical uses.

In comparison with other DNA-templated metal NPs, the synthesis of DNA-templated CuNPs is more convenient and significantly faster (Liu et al., 2018). DNA-templated Cu nanoparticles (NPs) exhibit great potential for instantaneous reaction, and facile integration with nucleic acid-based signal amplification and target-recognition strategies. This formation is associated with the clustering of Cu(0), which is produced by the chemical reduction of Cu(II) on the DNA backbones. Based on the quantum-confinement effects, CuNPs can be excitable by a λex at 340 nm to emit fluorescence signals with max λem at 600 nm for the *in-situ* production of fluorescent DNA-templated CuNPs in minutes. In addition, DNA-templated CuNPs are found to show large stokes shift that can help to reduce the background interference from the complicated biological matrixes. Hence, researchers investigated a dsDNA CuNPs-PCR method to rapidly detect the IS6110 DNA sequence of Mtb by *in situ* formation of a DNA-templated fluorescent CuNPs system (Tsai et al., 2019). With combination of smartphone-assisted image analysis to quantify target DNA, the detection instrument size/cost could be reduced and the portability was enhanced when compared with electrochemical nanobiosensor. More importantly, their results showed that this proposed method achieved a LOD of 5 fg/μl in analyzing clinical TB nucleic acid samples with a linear of 10–100 fg/μl. And using this nanobiosensing method, authors successfully distinguished nine TB-negative patients from three positive TB patients. In this manner, it becomes necessary to use this method for more large-scale clinical sample investigations. And in the future, it is possible to further avoid systemic variations induced by different smartphones as detectors through using a dongle/cartridge containing LED light source and photodetector with wireless transmission function. There is also another option, which can develop a cell phone app to perform control tests for results calibration. More interestingly, DNA target molecules from drug-resistant TB and multidrug-resistant TB can also be detected with this approach by simply using different DNA probes for diagnosis.

In addition to using varies kinds of nanoparticles, nanowires can also be introduced to develop TB nanobiosensors. A nanostructure platform was constructed by combining the gold electrode, nanowires of polypyrrole (nw-PPy), dendrimers PAMAM and ferrocene as well as DNA probe (Khoder and Korri-Youssoufi, 2020). The properties of nw-PPy (such as hydrophilic character and large surface area) significantly increased the electrochemical signals based on the outstanding electron transfer rate of 18 s^−1^, which allowed the enhancement of DNA sensing with a detection limit of about 0.36 atomolar without the use of amplification step. This biosensor was applied for the detection of genomic DNA of Mtb and mutated rifampicin resistant Mtb with high selectivity, which therefore show strong potentials for clinical sample detection. However, no direct evidences for the clinical sample analysis were presented in this work, which therefore still need further clinical sample validations of this nanobiosensor.

And to improve the detection efficacy, researchers developed many kinds of nano-biosensors based on the detection of TB DNA through electrode modification by functional nanomaterials (see Table 1). However, those diagnostic methods still need to be validated with clinical samples. To evaluate the sensitivity and specificity of this AuNPs-assisted colorimetric detection method, El-Samadony et al. (2019) designed a-case control study by analyzing the collected clinical sputum samples. DNA extracted from sputum samples were used for PCR analysis targeting IS6110 TB DNA. The results obtained by this TB nanogold assay could be observed by the naked eye just after 2 min of the addition of AuNPs to the test solution. Positive result could be defined as red color and negative result could be defined as red-to-violet color shift of the tested solution, which therefore allows very rapid and direct results for the tests. Their estimated TB nanogold assay showed higher performance than the combining of sputum smear microscopy (SSM) with chest X-ray (CXR), which indicated accurate and rapid detection for potential uses in clinic to improve accuracy of SSM and CXR. However, their verification experiment just involved a small sample size which therefore need more larger bacthes of clinical sample analysis. Some other developed new nanobisosensor based detection methods also did verification experiments by some TB clinical samples or even by adding the target markers into healthy patients’ serum, however, these strategies also need larger sizes of clinical sample analysis (Chen et al., 2017; Bai et al., 2019; Tsai et al., 2019).

**TABLE 1:** Comparison of the performance of different kinds of DNA nanobiosensors.

Modified electrode	Probe	Signal detection method	Linear range	LOD/Sensitivity	Reaction time	References (publication years)
FLG/GNus	ssDNA	SPR	—	24.5 fM	—	Prabowo et al. (2021)
FLG/GNrs	ssDNA	SPR	—	8.2 pM	—	Prabowo et al. (2021)
GCE-ZnO-AuNPs	ssDNA	DPV	2.5–250 pM	1.8 pM	45 min	Hatami et al. (2020)
Gold electrode/nw-PPy	(Mutated) *rpoB* gene	SWV	1 aM–100 fM	0.36 aM	30 min	Khoder and Korri-Youssoufi, (2020)
SPs-G-nano-C_60_/NG + CPs-bitoin-adivin-AuNPs/GCE + TOAB	*IS6110*	DPV	10 fM–10 nM	3 fM	4 h	Bai et al. (2019)
CuNPs	*IS6110*	Fluorescence stokes shift	10–100 fg/μl	5 fg/μl		Tsai et al. (2019)
3D graphene-GNrs	*IS6110*	EIS	10 fM–100 nM	—	—	Perumal et al. (2018)
Gold electrode/CPs + SPs/AuNPs/PAn-rGO	*IS6110*	DPV	0.1 pM-10 nM	50 fM	2.5 h	Chen et al. (2017)
SPCE/NH2-GO/CdSe QDs	PNA-DNA	DPV	10 pM-100 nM	0.895 pM	50 min	Mat Zaid et al. (2017)
Gold nanotubes array electrode platform	ssDNA	DPV	10–10^^5^ pg/μl	10 pg/μl	50 min	Torati et al. (2016)
CdSe QDs-SA/MCH/probe DNA/AuNPs/GCEs + *msp I*	DNA	SWV	10 fM–1 nM	8.7 fM	6 h	Zhang et al. (2015)
MWCNTs-PPy-PAMAM-Fc	*rpoB* gene	SWV	1 pM–100 nM	0.3 fM	60 min	Miodek et al. (2015)
Ru-GO/Fc-ssDNA	Mutated *rpoB* gene	ECL	0.1–100 nM	0.04 nM	50 min	Li et al. (2014)
GCE/rGO–AuNPs-PANI nano-composite	DNA	DPV	1 fM–1 nM	1.0 fM	4 h	Liu et al. (2014)

Note: PAn, means polyaniline; rGO, means reduced graphene oxide; SPCE, means screen-printed carbon electrode; NH2-GO, means amino group functionalized graphene oxide; QDs, means quantum dots; MCH, means 6-mercapto-1-hexanol; MWCNTs, means multiwalled carbon nanotubes; PAMAM, means poly (amidoamine) dendrimers; Fc means ferrocene; Ru-GO, means ruthenium (II) complex functionalized graphene oxide; DPV, means Differential pulse Voltammetry; SWV, means Square Wave Voltammetry; EIS, means Electrochemical Impedance Spectroscopy; ECL, means electrochemiluminescence.

In recent years, nucleic acid testing strategies have been widely developed and used worldwide under the COVID-19 epemdics, which also remind the potentials of nucleic acid testing strategies for other disease diagnosis. For the DNA analysis of Mtb, samples are very important issues as most clinical samples might didn’t have Mtb DNA contents. The most available and non-invasive samples for pulmonary TB analysis based on the DNA is the sputum sample, which requires DNA extraction procedures for further DNA analysis. Unlike the typical polymerase chain reaction (PCR) methods, most nanobiosensors don’t need amplification precodures and allow the direct analysis of DNA samples with shorten analysis time after the successful fabrication of nanobiosensors. But the times and costs used for the complicated nanobiosensor preparation should be a disadvantages compared with the PCR strategy. Moreover, the DNA from dead Mtb could also be detected by the DNA analysis methods, which therefore remind that the DNA detection method should act as auxiliary strategies for the current clinical diagnostic strategies.

## Detection of Mtb proteins by functional nanobiosensors

### Nanobiosensors targeting CFP10/ESAT6

Although DNA detection possesses the advantage of high sensitivity as well as high specificity, it requires preparation of DNA samples that needs special instruments, which cannot meet rapid diagnostic needs. Moreover, DNA detection could not effectively distinguish TB patients from latent TB patients. Therefore, some researchers pay more attentions to the protein detection that naturally secreted by Mtb, which allows the rapid diagnosis for the timely treatment of TB. To date, various kinds of proteins has involved in for target biomarkers of *Mtb*, such as the 6-kDa early secreted antigenic target (ESAT6) (Diouani et al., 2017), 10-kDa culture-filtered protein (CFP10) (Welin et al., 2015), *Mtb* protein 64 (MPT64) (Arora et al., 2015), the secreted protein antigen 85 complex B (Ag85B) (Saengdee et al., 2016), proline-proline-glutamic acid (PPE68) (Xu et al., 2012; Wang et al., 2020).

Comparative genomics analysis has revealed that the CFP10 and ESAT6 are encoded by the Rv3874 and Rv3875 genes, respectively. These genes are located in the region of difference-1 (RD-1) of the virulent Mtb genome but are absent in all strains of vaccine-BCG strains (Mahairas et al., 1996; Behr et al., 1999; Gordon et al., 1999; Aagaard et al., 2006). These two low molecular weight secretary proteins can bind with each other through the Van der Waals and hydrophobic interactions. Therefore, the formed ESAT6-CFP10 complex is a specific antigen for the virulent Mtb that can no be found in BCG (Vordermeier et al., 2001), in another words, prior BCG vaccinations in the patients would not induce false-positive results using CFP10 and ESAT6 as detection targets. In addition, both CFP10 and ESAT6 are secreted by Mtb at an early stage of infection (Renshaw et al., 2002). Hence, these two proteins can be used as early diagnostic markers of TB with the potent to distinguish between Mtb-infected and BCG-vaccinated groups (Weldingh and Andersen, 2008).

Similar to DNA detection, the pivotal part of nano-biosensor development for protein detection is biosensor modification. As early as in 2016, Noremylia’s laboratory has developed a G-interdigital electrode (IDE)/CFP10-ESAT6 aptamer/DNA-AuNPs based system as multi-channel piezoelectric quartz crystal (MSPQC) sensor to detect the combination protein of CFP10-ESAT6 (He et al., 2016). Here, the complementary DNA-AuNPs were modified onto the microelectrode with CFP10-ESAT6 aptamer functionalization to obtain the G-IDE/CFP10-ESAT6 aptamer/DNA-AuNPs probe. CFP10-ESAT6 aptamer could interact with the CFP10-ESAT6 protein specifically to form complex on the surface of electrode, which would result in the fell away of the DNA-AuNPs fragments from the electrode surface. These changes can be captured by the IDE-MSPQC sensor with high sensitivity. Other kinds of bacterial like *M.smegmatis*, *S.aureus*, *E.coli*, and *E.faecaliss* were also used for analysis by the above sensor to show frequency shift lower than exceed 15 Hz, whereas it reached 73–121 Hz in Mtb detection, indicating the high selectivity of the proposed sensor. This approach was specific, more sensitive, and expected to become a valuable analysis tool for the early detection of M. tuberculosis in clinical sample. However, the detection times of this method need up to 96.3 h, which couldn’t achieve the need of rapid detection. Moreover, this nanobiosensor was also not used for clinical sample analysis, which requires further validations of this strategy in clinical samples.

Additionally, Noremylia and co-workers developed a (CdSe-ZnS QD/silica NPs (SiNPs)/SPCE)-modified electrode as nanobiosensors to detect CFP10-ESAT6 fusion protein (Mohd Bakhori et al., 2019). The obtained electrode could be linked to the biocatalytic interactions for the enzyme catalase through specific antigen-antibody binding by means of producing a DPV current. The developed method indicated a linear range of signals towards CFP10-ESAT6 in different concentrations with a *R* ^2^ value of 0.9937, yielding a LOD of 0.15 ng/ml and good reproducibility for the targeted analyte. However, this nanobiosensor is also not applied for the more complicated clinical sample analysis. And they further found that DPV responses for both BSA and MPT64 were more than half of that for CFP10-ESAT6 detection, which might be attributed to that the modification of the electrode by CdSe-ZnS QD/SiNPs/SPCE could increase the effective surface area of the electrode to lead the peak current. These results showed that the selectivity and specificity of their obtained nanobiosensor are not strong enough as other kind of proteins in the complicated clinical samples might affect the accuracy of the results. Thus, the selectivity and specificity of the proposed nanobiosensor remain to be further improved, which would be the most critical issue for their following clinical sample researches.

Researchers also designed a CFP10-ESAT6 targeted aptamer-graphene (GP)-PAn-screen-printed gold electrode to detect the CFP10-ESAT6 fusion protein (Azmi et al., 2021). By interacting with the CFP10-ESAT6 antigen complex, the specific aptamer could capture the targets and the antibody conjugated Fe_3_O_4_/Au-MNPs was used for sandwich format. Based on these properties, CFP10-ESAT6 antigen can be effectively detected by the differential pulse voltammetry (DPV) technique. Unlike the common electrochemical detection using potentiostat conducted in the laboratory, this portable reader is small in size, easily handled, and can be used for on-site monitoring. In addition, the detection times for this method was only 55 min (including the time of sample pretreatment), which might allow very rapid diagnosis. This study involves the utilization of iron/gold core/shell magnetic nanoparticles (Fe_3_O_4_/Au MNPs) which would be strongly attracted to the Ab. The incorporation of gold (Au) coating on a magnetic core could combine the advantages of high chemical stability/biocompatibility of gold and high magnetic separation efficiency of Fe_3_O_4_. This strategy allows the detection of CFP10-ESAT6 antigen complex at a concentration range of 5–500 ng/ml to show a LOD of 1.5 ng/ml. Moreover, the specificity would not be easily disturbed by other proteins like BSA, MPT64, and human serum. Mohd Azmi et al. (2021) also proposed sandwich-type immunosensor with a portable, rapid, and simple properties for the detection of Mtb in sputum samples by targeting CFP10-ESAT6 complex. This strategy indicates almost 100% sensitivity and 91.7% specificity compared with the standard methods (culture and smear microscopy) for Mtb analysis in the sputum samples, which suggested the potential of the proposed device for practical screening. In clinical sample detection, these two methods showed positive DPV signal changes in TB positive samples and negative changes in negative samples, showing absolutely 100% specificity and sensitivity (Azmi et al., 2021; Mohd Azmi et al., 2021). However, more works need to be done on larger sizes of clinical samples to make sure the applicability of this nanobiosensors for clinical uses. And further investigations are still needed to be done, such as the improvement of the stability, sensitivity and robustness of the sensor.

In addition to biosensor modification, researchers also tried to develop a label-free method for biosensor preparation to detect the CFP10-ESAT6 fusion protein, which might allow more convenience for TB diagnosis (Sun et al., 2017). Metal-organic framework (MOF-rGO) was doped onto the reduced graphene oxide, which were further deposited onto a glassy carbon electrode (GCE). Platinum/gold core/shell (Pt@Au) based nanosystem was prepared to assemble thiolated ESAT-6 binding aptamer (EBA) on a modified electrode and to further amplify the response to TB. Firstly, P-MOF-rGO nanocomposite was employed to enhance the loading of electroactive toluidine blue (TB). Then, Platinum aurum core shell (Pt@Au) nanoparticles provided abundant active sites for the immobilization of thiolated ESAT-6 binding aptamer (EBA) and further amplified the electrochemical response signal of TB. After the specific recognition between ESAT-6 antigen and EBA, significant change of electrochemical signal indicated that the aptasensor was successfully constructed. In that method, GNrs contained the fusion protein of CFP10-ESAT6 was directly incubated with clinical serum samples to detect CFP10-ESAT6. After incubated with collected serum samples for 2 min, the biosensor indicated changes in the SPR properties of GNrs due to the specific antigen-antibody interactions. According to the detection results, the sensitivity was 79% and the specificity was up to 92%, which indicated the potentials of this method to be a valuable tool for diagnosis of TB.

There are also publications about ESAT6 or CEP10 detection separately. Li et al. (2018a) developed an electrochemical nanoaptasensor to measure ESAT-6 protein. In their strategy, GCE electrode was modified by rGO-metal-organic framework complex (MOF-rGO) and Toluidine Blue, which could enlarge the surface area and facilitate the electron transferation. Later, thiolated ESAT-6 binding aptamer was conjugated onto (EBA)-platinum/gold core/shell (Pt@G) nanoparticles complex, which were then deposited into the above modified GCE electrode. The electrochemical signal was originated from the changes of Toluidine Blue based on the specific interactions between EBA and ESAT-6. Results demonstrated that the developed electrochemical nanoaptasensor had a linear response in 0.1 pg/ml to 200 ng/ml ESAT-6 concentration range with a LOD of 0.033 pg/ml. Compared with the detection result of the same concentration of other interference molecules, like glucose, CFP-10 and BSA, the specific recognition and interaction of the targeted biomolecule and nanoaptamer led to significantly decreased electrochemical signals, indicating the high selectivity of the proposed nanobiosensor for ESAT-6 detection. In another study, a new and one-step approach based nanobiosenser was designed to measure ESAT-6 (Wang et al., 2018). Within that detection system, AuNPs would change its colors from red to blue followed by the salt-induced aggregation if anti-ESAT-6 antibody was pre-mixed with ESAT-6, whereas the color did not change in control system (CFP-10). The red spectral shift could be visualized or measured by a UV-spectroscopy with a LOD value of 1.25 pM. Nevertheless, the sensitivity was easily influenced if the concentration of antibody and salt were not optimized. Further, this assay can be expanded with a small molecule complexed with an appropriate antibody to be possible for potential point-of-care diagnosis. This strategy could definitely work in proteins and peptides with small molecular weight, while the large proteins might introduce variations due to the amino acid charges.

Similarly, nanobiosensors can also be designed to detect CFP-10 alone. Researchers used Fe_3_O_4_@Ag/GPQDs to modify the GCE electrode for the formation of a sandwich complex on the electrode surface, which could be used for DPV signal collection. This nanoscale sensing platform allows synergetic electrochemical performance based on the properties of the three nanomaterials: Firstly, Fe_3_O_4_ nanomaterials could increase the surface/volume ratio of the nanobiosensor; then, Ag nanomaterials could enhance the electrical conductivity of the nanobiosensor; and GQD could load more anti-CFP-10 antibody onto the nanobiosensor. But the electrochemical nanoimmunosensor showed a linear range (5–5.0 × 10^5^ ng/ml) and a LOD value of 0.33 ng/ml, which was a relatively low sensitivity comparing with other nanobiosensors (Tufa et al., 2018). These results suggest that this proposed nanobiosensor with good performance and high selectivity might also be used for the analysis of other kind of biomolecules of Mtb. However, how to extend this strategy for the much more complicated clinical sample analysis remain a big challenge.

The electrochemical nanobiosensor detection method developed by Azmi et al. (2021) was also used to detect CFP-10 using CFP-10 antibody. However, compared to the results for the fusion CFP-10-ESAT6 protein detection, analysis of CEP-10 alone by this method didn’t show similar good linear range (20–100 ng/ml) and LOD (15 ng/ml) value (Mohd Azmi et al., 2018). CFP-10 aptamer can also be designed to capture CFP-10 antigen (Li et al., 2019b). CFP-10 is specifically captured by its aptamer and then induces a DNA cross-linking click reaction, the release of CFP-10, and an amplification cycle of repeated CFP-10 release. High occurrence of CFP-10 would cause the more CFP-10 aptamer strands on the gold electrode surface to expose their 5′ overhang and to hybridize with the DNA complexes linked to the AuNPs. Consequently, large amounts of AuNPs, which were loaded with lots of quadruplex DNA motifs, could be bound onto the electrode surface to remarkably enhance the electrochemical signal. This assay shows high selectivity toward CFP-10 antigen due to its specific interactions with the aptamer. However, based on the clicking chemical reactions for the target proteins and the G-quadruplex DNA motifs, this assay indicated a very low detection limit against the targeted CFP-10 antigen. In optimal conditions, this method demonstrated linear range of 0.01–100 ng/ml and LOD value of 10 pg/ml for CFP-10 analysis. In sputum sample analysis, their nanobiosensor indicated that the DPV signal changes in TB patients was much higher than the DPV signal changes of healthy volunteers.

## Nanobiosensors targeting MPT64

MPT64, a 24-kDa protein only secreted by *Mtb* in the early/middle growth stage (Yin et al., 2013; Jiang et al., 2014), can also be utilized for Mtb detection (Dahiya et al., 2020). The aptamers that can bind specifically with MPT64 have already been reported (Qin et al., 2009). Hence, Li’s team developed a voltammetric nanobiosensor based on two aptamers for MPT64 detection using AuNPs and Zr(IV)/terephthalate MOF (metal-organic framework) to modify gold electrode (Li et al., 2018b). Firstly, amino-modified Zr(IV) MOF was synthesized and was then loaded with AuNPs and aptamers, which was then used for nanoprobe fabrication by casting horseradish peroxidase onto the nanomaterials. These two different aptamers loaded onto the gold electrode could show synergistic effects for MPT64 binding with highest differential pulse voltammetry when the aptamer ratio is 1:1. This method showed a wide linear response range (0.02–1000 pg/ml) and a 10 fg/ml detection limit. Moreover, DPV results demonstrated the fabricated aptamer-based nanobiosensor had satisfactory selectivity for MPT64 from BSA and CFP10-ESAT6. The biosensor do exist some defects such as it still has some difficulty on clinical application and the modification process of electrode will be affected by various factors.

Guo’s team also developed an aptamer-based voltammetric nanobiosensor for MPT64 detection by using synergistic signal amplification strategy based on a novel GO@Fe_3_O_4_@Pt nanocomposite (Gou et al., 2018). Based on this strategy, the constructed nanobiosensor showed a wide linearity range (5.0 fg/ml to 1.0 ng/ml) and a LOD of 0.34 fg/ml with less than 4 h of detection time. More importantly, when the nanobiosensor was used for clinical serum samples analysis from TB patients to show high current signals, whereas the signal was negligible in healthy or non-TB group. These results indicated that this proposed nanobiosensor had strong potentials to provide the basis for rapid clinical diagnosis of *Mtb* infection, although a larger scale clinical sample analysis are still needed. In addition, some researchers introduced a sandwich-like electrochemical aptasensor based on carbon nanocomposite formed by nitrogen-doped carbon nanotubes, fullerene nanoparticles and grapheme oxide, which introduced larger specific surface area to obtain high conductivity and electroactive property for redox biosensing (Chen et al., 2019). The above nanocomposite was further modified with AuNPs to bind with the MPT64 antigen aptamer, which could generate effective amplification of the signals. Moreover, polyethyleneimine (PEI) modified MOF was used to capture Au–Pt nanoparticles (Au@Pt), which significantly accelerated electron transfer and increased the immobilization efficiency of aptamer. The AuNPs. The AuNPs containing MPT64 aptamer were immobilized in the C60NPs-N-CNTs/GO and conductive polyethyleneimine (PEI)-functionalized Fe-based metal-organic framework (P-MOF), to form a sandwich composite with the MPT64 antigen. The proposed aptasensor showed a wide linear range (1 fg/ml–1 ng/ml) with a LOD as low as 0.33 fg/ml. Similarly, the biosensor also indicated excellent sensitivity and specificity, but the gap of current response between TB serum specimens and healthy controls was a little bit small (about 2-fold), which need to be further confirmed by more clinical sample analysis.

In addition, AuNPs-C_60_-PAn was also used as a kind of nanoprobes for redox analysis by detecting MPT64 antigen with high sensitivity, which showed linear range of 0.02–1,000 pg/ml and a LOD value of 20 fg/ml. This strategy showed current response was up to 15.58 μA when the concentration of MPT64 in clinical samples was as low as 0.2 pg/ml, whereas the current response in negative control was almost negligible (Bai et al., 2017). More importantly, this biosensor also showed high specificity and sensitivity for the detection of MPT64 antigen in serum samples, which therefore allowed the ability to distinguish TB patients from healthy donors.

However, the synthesis processes of nanomaterial for these electrochemical nanobiosensors detection method, especially for the nanobiosensor fabrication processes, were relatively complicated and the detection sensitivity, efficacy and selectivity also need to be further confirmed by more clinical sample analysis. Conceivably, these nanobiosensor assays for MPT64 detection demonstrate large potentials in providing new evidences for the rapid diagnosis of *Mtb* infection.

### Nanobiosensors targeting Ag85B

Ag85 proteins are one of the most immunogenic antigens obtained from *Mtb* culture filtrate (Malen et al., 2008). As one of the representative secreted proteins in Ag85 proteins, Ag85B plays critical roles in the physiology of Mtb and has been proposed as a promising marker for potential *Mtb* diagnosis (Che et al., 2016; Zhang et al., 2018). A sandwich assay detecting Ag85B combining GNrs) and silica coated QDs (SiQDs) was produced by Eun’s team (Kim et al., 2017). In this nanobiosensor, genetically engineered recombinant antibody was bound onto the surfaces of GNrs and SiQDs respectively without any surface modification. These two biocomplexes showed quenching fluorescence intensity in the presence of the target antigen through a sandwich assay. At high Ag85B concentrations, the distance between GNrs and SiQDs got closer, which thus led to the decrease of fluorescence through fluorescence resonance energy transfer (FRET) with an assay response range of 10 pg/ml–1 ng/ml and a LOD of 13 pg/ml. When compared with some interfering proteins like BSA or CFP10, the obtained fluorescence intensities were almost the same with the fluorescence intensities obtained from control samples, whereas Ag85B led to significant decreases of fluorescence intensity, indicating the specificity and selectivity of the proposed method. Moreover, the recovery experiments showed 92%–104% through measuring different concentrations of spiked clinical urine samples. Although the authors stated that they developed a highly sensitive and selective nanobiosensor for better diagnosis of TB by Ag85B-expressing Mtb detecting compared with the existing methods, no clinical samples were used for analysis by this nanobiosensor. More works are needed to compare this nanobiosensor with the existing clinical strategy on a large scale clinical samples.

By analyzing the above Mtb-specific proteins, the sensitivity and accuracy of TB diagnosis can be significantly enhanced by combining with some other diagnostic tools. As these target proteins can be secreted into environments and further transfered into different parts of body, different kinds of samples, such as blood samples (serum or plasma) and sputum samples, can be applied for protein analysis. Due to the complicated protein contents in serum or plasma samples, the specificity of nanobiosensors is of vital importance for Mtb-specific protein analysis. However, there are some target proteins that might be disturbed by the TB vaccine-BCG vaccination, such as Ag85b, which is both expressed and secreted by BCG and Mtb. Thus, developing nanobiosensors that can simultaneously detect different kinds of Mtb-specific proteins might be an attractive strategy for more sensitive and accurate TB diagnosis.

### Detection of cytokines by nanobiosensors

Cytokines, some small peptides or glycoproteins, are synthesized and secreted by immune cells and certain non-immune cells. Cytokines have a variety of biological functions, such as regulating cell growth, differentiation and maturation, immune response, inflammation, wound healing, tumor growth and function maintenance (Pourgholaminejad et al., 2016; Wang et al., 2021). Host cytokine levels are different at different stages of Mtb infection and may serve as markers of Mtb infection (Sudbury et al., 2020).Many teams evaluated candidate cytokines as biomarkers to distinguish LTBI infection from active TB, and their results were generally associated with IL-10, IFN-γ, IP10, IL-2, TNF-α and VEGF5, with IFN-γ being the most popular cytokine by far (Gong and Wu, 2021).It has been mentioned above that the existing cytokine detection is not real-time, expensive and difficult to operate. However, the development of nanobiosensors provides a way for its development (Perez et al., 2022). And as the most important cytokine that involved in TB infection, IFN-γ detection will be taken as an example to summarize the current status of nanobiosensors in the diagnosis of TB.

In a fluorescence nanobiosensor, when biometric elements or targeted biomolecules are labeled by fluorescent labels, the fluorescence intensity is used to reveal how strong about the interactions between the biometric molecules and the target biomolecules. Chen et al. (2022) developed a novel aptamer sensor for IFN-γ analysis using PPI-CE CPNs (cerium pyrophosphate coordination polymer nanoparticles) as a signal reporter molecule and using double-stranded DNAs as probes. The sensor is implemented by spatially regulating polymeric extension of terminal deoxynucleotide transferase (TdT) as well as the selective recognition of PPiCe CPNs. This method allowed a detection limit of 0.25 fg/ml with a linear range of 1–100 fg/ml and high specificity. This IFN-γ biosensing method was further validated in 57 clinical samples, which were proved by some current clinical TB diagnostic methods, indicating the ability of this nanobiosensor as a more sensitive tool for potential early diagnosis of TB. However, this method can’t allow the direct naked-eye visual readout analysis. This, ultrasensitive IFN-γ quantitative method is expected to effectively reduce the serum stimulation culture time, and provide a novel tool for the early and rapid diagnosis of TB.

In another study, biosensors based on hairpin structures of oligonucleotides, single-stranded DNA-binding proteins (SSB), copper nanoparticles (CuNPs), and silica nanoparticles coated with Streptavidin were synthesized (Taghdisi et al., 2017). The presence of double-stranded DNA (dsDNA) regions and polythymidine (T) in hairpin structures of oligonucleotide, SSB, and SNP streptavidin results in highly selective and sensitive IFN-γ assays. Here, the hairpin structure of the used oligonucleotide allowed high specificity of the proposed aptamer nanobiosensor. The addition of IFN-γ could disassemble the structure of oligonucleotide to result in a weak fluorescence signal. And if no IFN-γ was added, no structure changes would happen in oligonucleotide, which would result in stronger fluorescence signal. In optimal cases, IFN-γ was detectable at concentrations as low as to 1 pg/ml with a linear range of 10–4,000 pg/ml. The above method was further used in spiked human serum samples for IFN-γ analysis with recovery value about 92.52%–98.32%, which indicated the promising potential of this method in real biomedical analysis. However, no samples from TB patients were analyzed to test the ability of this nanobiosensor for potential TB diagnosis. Thus, further investigations about clinical samples from TB patients and health controls should be applied for this proposed nanobiosensors.

In addition to the above nanibiosensors, other teams have also designed different fluorescence aptamer sensors with excellent characteristics such as high sensitivity and strong specificity (Wen et al., 2018; Wen et al., 2019), as shown in Table 2. Both of these two works indicated a great potential in IFN-γ detection of real samples by these nanobiosensors with high selectivity, efficiency and stability. However, it is still need to further confirm the potentials of these nanobiosensors for natural human serum sample analysis, especially for their ability for the serum analysis from TB patients.

**TABLE 2:** Comparison of the analytical performance of nanobiosensors for the analysis of cytokines.

Biosensor devices	Active analytical layer	Linear range	LOD/Sensitivity	Reaction time	References (publication years)
Fluorescent aptasensor	PPi-Ce CPNs/Cu NPs	1–100 fg/ml	0.25 fg/ml	—	Chen et al. (2022)
Fluorescent aptasensor	Oligonucleotide-SNPs-streptavidin-SSB-CuNPs	10–4,000 pg/ml −	1 pg/ml	55 min	Taghdisi et al. (2017)
Fluorescent aptasensor	Fe3O4-aptamer 1/IFN-γ/aptamer 2/dsDNA	0.169–169 × 10^5^ pg/ml	2.95 × 10^–3^ pg/ml		Wen et al. (2018)
Fluorescent aptasensor	Fe3O4-aptamer 1/IFN-γ/aptamer 2-PBiB-pBIEM	3.38 × 10^−5^–8.44 × 10^5^ pg/ml	3 × 10^−3^ pg/ml		Wen et al. (2019)
Electrochemical aptasensor	*Au-Gra/dsDNA/*IFN-γ/[Ru(NH3)6]^3+^/SP-Au@Fe3O4		0.003 ng/ml	1 h	Liu et al. (2015)
Electrochemical aptasensor	*{H2/H1} n/IFN-γ/CP/*(*+*)*AuNPs/Nf*	50 fM–3.0 pM	16.3 fM	200 min	Miao et al. (2017)
Electrochemical aptasensor	*GE/aptamer/IFN-γ/Exo I and Exo III*	16.9–8.44 × 10^5^ pg/ml	11.8 pg/ml	—	Li et al. (2019b)
Electrochemical aptasensor	*Au IDE/ACP/HDT-MCH*	375–1860 pg/ml	195 pg/ml	35 min	(Ding et al., 2017)
Electrochemical DNAzyme biosensor	T-DNA/MCH/CP/AuNCs-Gr@ZIF-8/GCE	0.0169–844 pg/ml	0.01 pg/ml		Bao et al. (2019)
DNA photoacoustic nanosensor	Streptavidin-coated SPR chip-IFN-γR2-IFN-γR1-IFN-γ	10–2,000 pg/ml	KD1 2.8 × 10^6^ pg/ml KD2 5.3 × 10^6^ pg/ml		Morales et al. (2019)
Electrochemical immunosensor	AJP graphene IDE-anti-IFN-γ-BSA-Tween-20-fish gelatin	100–5,000 pg/ml	25 pg/ml	60 min	Parate et al. (2020)

In addition to fluorescent aptamer sensors, there are electrochemical sensors, which are sensing devices that couple biometric elements to electrode sensors. The transducer then converts biometric events into electrical signals. A novel electrochemical aptamer sensor for IFN-γ detection was developed based on exonuclease-catalyzed target recycling and TdT mediated cascade signal amplification (Liu et al., 2015). Previously hybridized double-stranded DNA (trapping probe hybridized with complementary IFN-γ junction suitable body) was immobilized on Au nanoparticle-graphene nanocomposite (Au-GrA) membrane modified electrodes. When IFN-γ is presented in the tested samples, there would form aptamer-IFN-γ complexes, which would further result in the release of aptamers from the dsDNA. The aptamer is selectively and specifically digested using exonuclides to release IFN-γ for target recovery. A large number of probes with single-stranded capture ability would form and result in hybridization to introduce the probe labeled Au@Fe3O4 system. The structures of long single-stranded DNA can be formed by catalyzing the labeled probe sequence by terminal deoxynucleotide transferase (TdT). Finally, the electron medium ruthenium (III) hexamines chloride ([Ru(NH3)6]^3+^) can interact with DNA to generate a strong electrochemical signal for quantitative measurement of IFN-γ. And by using Au-Gra as substrate, the proposed aptamer nanobiosensor shows wide detection liner range with a very low detection limit of 0.003 ng/ml. And taking the advantages of highly effective amplification, this aptamer nanobiosensor is very easily to be operated without complicated labelling procedures. Additionally, with valid signal amplification ability and simple structures, the above aptamer nanobiosensor uses might allow the further development of more versatile methods for other biomolecule detection.

Additionally, Miao et al. (2017) also prepared a novel Iridium (III) complex for sensitive electrochemical detection of IFN-γ. Firstly, the electrode surface was immobilized by Nafion (Nf) and (+)AuNPs composite films, which were then conjugated with a IFN-γ interaction chain contained capture probe by Au-S binding. After addition of IFN-γ, the ring-shaped stem structure of CP is opened, and the newly exposed “sticky” region of CP is subsequently associated with DNA hairpin 1 (H1), which in turn opens its hairpin structure for hybridization with DNA hairpin 2 (H2). HCR (hybrid chain reaction) occurs between H1 and H2 to produce polymeric double-stranded DNA (dsDNA) strands. At the same time, the Iridium (III) complex can interact with the groove of the double-stranded DNA polymer to produce a strong current signal proportional to the concentration of IFN-γ. Based on this novel nanobiosensor, the detection of IFN-γ can be achieved with high sensitivity and a detection limit of 16.3 fM. Compared with the enzyme-assisted signal amplification technique, HCR could achieve high robustness and stability of the nanobiosensor without the use of enzymes. Moreover, iridium (III) complex in the nanobiosensor could interact with the unlabelled dsDNA polymers, which allows the detection of IFN-γ with low-cost. In addition, other teams have also designed different electrochemical sensors with excellent features such as high sensitivity, high specificity, and strong (Ding et al., 2017; Li et al., 2019a; Bao et al., 2019; Morales et al., 2019; Parate et al., 2020), as shown in Table 2.

To be honest, cytokine detection of IFN-γ is not a highly specific method for TB diagnosis as IFN-γ is widely involved in different diseases, which means that it is very difficult to distinguish TB from other diseases based on IFN-γ detection. Thus, it is very important to explore more specific strategies for TB diagnosis, which might be achieved by the simultaneous detection of several different kinds of cytokines.

Nanomaterial-based biosensors can also be developed for multiple cytokines detection at the same time. For example, a team synthesized a graphene immunosensor based on aerosol jet printing (AJP) that can monitor two different cytokines: IFN-γ and IL10 (Parate et al., 2020). A 40 μm wide interfinger electrode (IDE) was printed on a polyimide substrate using graphene-nitrocellulose ink. By annealing the IDE in CO_2_, ROS (reactive oxygen species) can be introduced onto the graphene surface to serve as a chemical handle that can covalently and effectively attach IFN-γ/IL10 antibodies onto the surface. The resulting AJP electrochemical immunosensor can monitor serum cytokines with a sensing range for IFN-γ of 0.1–5 ng/ml and for IL-10 of: 0.1–2 ng/ml, which allows potential TB diagnosis. Moreover, these biosensors are mechanically flexible with minimal change in signal output after 250 bending cycles over a high curvature (*Φ* = 5 mm). Hence, this technology could be applied to numerous electrochemical applications that require low-cost electroactive circuits that are disposable and/or flexible. The combination of IL-10, IP-10, and IL-4 can distinguish tuberculosis patients from latent tuberculosis patients, with sensitivity and specificity of 77.1% and 88.1% (Korma et al., 2020), respectively. The combination detection of IL-1α, IP-10, McP-1, TNF-α, and IL-10 was also shown to be excellent in the potential diagnosis of LTBI with high sensitivity and specificity (Luo et al., 2019). This also provides more directions for the subsequent development of nano-biosensors.

Up to now, the low contents of specific markers in human serum still dramatically restricted the diagnosis of LTBI. Yang’s group have recently introduced several different methods based on nanobiosensors for potential LTBI diagnosis. Firstly, a novel ECL-biosensing platform was developed for the detection of multiple LTBI markers, including IFN-γ and IL-2 (Zhou et al., 2017b). This proposed ECL-sensing platform allowed the accurate and specific detection of IFN-γ and IL-2 in human serum with high sensitivity, which provided a valuable protocol to develop fast and precise diagnostic strategy by nanobiosensing of LTBI samples. Moreover, they also tried to develop more specific detection method based on the simultaneous detection of three different kind of cytokines by fabricating potential-resolved ECL nanoprobes (Zhou et al., 2017a). The AuNPs and magnetic beads were conjugated with luminol carbon quantum dots and CdS quantum dots, followed by the immobilization of IFN-γ, TNF-α and IL-2 antibody onto indium tin oxide electrode as a nanobiosensor system for LTBI marker capture. The obtained ECL immunosensor provides an effective and high specific approach for the simultaneous detection of IFN-γ, TNF-α and IL-2 in human serum, which might be beneficial to facilitate more accurate and reliable clinical diagnosis for LTBI. In addition to the above electrochemiluminescence method, they further introduced a method for continuous monitoring of multiple cytokines using nanobiosensors based on quartz crystal microbalance (QCM) detection (Zhou et al., 2019). Although QCM is able to detect the mass change in the nanogram range, it is still limited in the detection of low-level antigen. In this work, mass signal amplifier-silver nanoparticles acting were conjugated with specific antibodies to form a kind of novel mass nanoprobes, which could increase the loaded mass on the surface of QCM. Hydrogen peroxide can oxidatively dissolve the prepared nanoprobes, which could avoid the steric hindrance of the probes. This method could be used to monitor IFN-γ, TNF-α, and IL-2 serially, thus providing a novel strategy for real-time monitoring of multiple LTBI related cytokines. Compared to MQCM, the above QCM strategy could effectively avoid the acoustic interference and simplify the instrumental setup procedures for sensitive and accurate detecting of multiple cytokine-associated LTBI biomarkers.

There are also clinical TB diagnostic methods that targeting the cytokine analysis, such as the IGRAs test, which detects the IFN-γ releases with few cross reactions with non-Mycobacterial or BCG infections. But it is worth to note that this method might also introduce false results in some subjects with inherent high IFN-γ production. Thus, it is a trend to develop new methods for multiple cytokine analysis, and among the current techniques, nanobiosensing techniques provide strong potentials for multiple cytokine analysis in potential TB diagnosis. But how to screen new Mtb-specific cytokines as new targets for analysis combining with IFN-γ remains a big challange. In theory, the direct analysis of plasma or serum cytokines of patients without antigen stimulation would be more easily to be disturbed by other diseased conditions. Thus, the development of new nanobiosensors should also be directed to detect the cytokine released from the PBMC of patients upon Mtb antigen stimulation.

### Direct whole cell detection of Mtb by nanobiosensors

In addition to DNA, secreted proteins and cytokines, researchers also introduced whole cell detection method for *Mtb* diagnosis. Teresa and co-workers used a magnetoresistive (MR) biosensors to detect BCG bacteria with magnetic nanoparticles (MNPs), functionalized with specific antibodies (Abs) and bioconjugated with BCG bacterial cells through three steps: A) capture Abs and control Abs were attached to the surface of MR-biochip, respectively; and then a baseline MR signal was registered; B) a immunoassay following a sandwich format labeled by MNPs and functionalized with polyclonal biotin was conjugated with anti-*Mtb* detection Abs to bioconjugate with the target bacterial cells; C) the non-bound MNPs were then washed out and the signals from labeled targets could be recorded. It is worth to note that the BCG binding specificity is not 100%, but there are significant differences for the MR voltages between positive sample and negative control (Barroso et al., 2018). Their results showed significant signals during sample detection with an estimated LOD value of 10^4^ cells/ml. A similar LOD detectability of 10^3^–10^4^ cells/ml can also be obtained by using magnetic barcode for nanobiosensor development in sputum sample detection (Liong et al., 2013).

In addition, Zhang et al. (2019) also developed a MSPQC sensor system for rapid detection of *Mtb*. The electrochemical sensor contains an H37Rv aptamer that were sequentially hybridized with three designed AuNPs−DNAs. When H37Rv was presented in the detected samples, it could specifically bind with the specific aptamer, which would lead to the release of AuNPs−DNA from the electrode and the non-conductive complex of aptamer and bacteria would replace the conductive layer of the electrode for MSPQC system detection. The frequency shift values of interfering bacteria, such as BCG, *Pseudomonas aeruginosa* and *S. aureus*, were not more than 25 Hz, whereas the frequency shift values of H37Rv was up to 236 Hz. Therefore, this AuNPs-DNA based and H37Rv aptamer conjugated MSPQC sensor can distinguish pathogenic from non-pathogenic bacteria, indicating the high selectivity and specificity. Taking L-J slant culture method as gold standard method, the sensitivity of this proposed nanobiosensor was 91% and the specificity was 90% in clinical sample detection. However, the detection time of the proposed sensor was only 2 h with a LOD of 10^2^ cfu/ml. It is very useful to shorten the 21 days detection time for L-J slant culture method to 2 h. However, the low sensitivity (LOD of 10^2^ cfu/ml) makes it very difficult to be used for real clinical sample analysis from TB patients as most samples from TB patients showing lower CFU than 100. Therefore, more works are needed to further improve the sensitivity of this nanobiosensing strategy.

In addition to the measure of electrical signal, magnetic nanoparticle based colorimetric biosensing assay (NCBA) can also be developed to detect acid-fast bacilli (AFB). Among that method, MNPs were coated by glycan, which allowed the effective capture of *Mtb* by glycan-glycoprotein interactions that didn’t require the use of the much more expensive antibodies or aptamers. The detection procedure was greatly simplified when compared to other signal detection methods such as electrical and fluorometric signal. More importantly, the detection results based on NCBA method showed 100% agreement with the results obtained by Xpert Mtb/RIF for all 500 tested samples, however, with much shorter time (10–20 min) and much cheaper costs (almost $0.10 per test), which might be more easily used for TB diagnosis in some developing and undeveloped countries (Abubakar et al., 2013). Blakemore et al. (2010) found that the lowest detection limit of Xpert system’s was nearly 131 CFU/ml. However, there is a disadvantage that this method can’t be used to distinguish pathogenic AFB from non-pathogenic AFB, which urges the authors to further improve the selectivity of this nanobiosensors.

In theory, the detection of whole cell of Mtb requires the existence of Mtb in the anayzed samples. Thus, the clinical analysis of whole cell of Mtb always performed in the sputum from potential pulmonary TB, such as the Mycobacterial culture analysis of Mtb in clinic. We need to know that there indeed exist potential biosafty issues for the use of sputum samples with live Mtb inside, which therefore requires strict laboratory conditions with high biosafety levels. Therefore, unlike the analysis of DNA, proteins or cytokines in the blood samples, the whole cell of Mtb analysis from sputum samples need to be strictky done in BSL-3 or at least BSL2+ labs. If new nanobiosensors are developed for whole cell of Mtb analysis, these nanobiosensors and their corresponding detecting instruments should also be set in the high biosafety labs, which might introduce more costs and biosafet issues.

## Perspectives and conclusion

As one of the most urgent public health issues, TB causes millions of deaths every year, ranking above AIDS. Thus, how to control the TB epidemic remains a big challenge. For rapid and effective therapy of TB patients, the diagnosis of TB is the most important issues, which therefore requires rapid and accurate TB diagnosis method. However, the current methods for TB, latent TB or drug-resistance TB diagnosis is not sensitive, rapid and accurate enough, which urges us to develop more sensitive, rapid and effective diagnosis strategy.

Taking the advantages of outstanding physical, chemical and biological properties, nanomaterials have been proved to show promising potentials for novel nanobiosensor construction. Here, we summarized the current progress of nanobiosensors for potential TB diagnosis application. Due to the unique properties of TB infection, most of the current diagnostic method based on nanotechnology is focused on the detection of Mtb components, such as the DNA and proteins released from Mtb. The positive signals for the nanobiosensor indicate the presence of DNA and proteins from Mtb in the sample and also indirectly indicate the infection of the sample sources. The detection of the DNA and protein of Mtb by nanobiosensor is much more specific for TB diagnosis than that of cytokine detection. Whole cell detection of Mtb is also more specific than cytokine-based detection methods. This is caused by the fact that cytokines are always involved in different diseases, which means that they are not specific for TB. Therefore, we prefer to suggest that the cytokine detection should act as a ancillary method combining with other methods, which would allow more accurate diagnosis of TB.

Another important issue is that the sample for analysis is also critical for the clinical application of nanobiosensors. The detection of the DNA contents and proteins of Mtb, or the detection of cytokines allow the analysis of some very easily obtained samples, such as serum, plasma or blood cells. However, the detection of whole cell of Mtb requires the samples containing Mtb, such as the alveolar lavage fluid or the tissues from the infection site. It is widely known that the tissue samples and the alveolar lavage fluid samples are much more difficult to be obtained. Additionally, the samples with Mtb inside are very dangerous infectious samples that should be carefully treated in specific labs, such as BSL3 labs or BSL2 labs with strict controlled conditions. Thus, although the whole cell detection method could provide direct evidences for the presence of Mtb in samples, it is still very difficult to be popularized in hospitals. It is worth to note that the sample collection and storage strategies are also very important for the rapid, accurate and sensitive analysis of TB by nanobiosensors. As different sample collection and storage strategies might result in different detection results, developing standardized sample collection and storage protocols should also be recognized as an important part for the furture clinical application of nanobiosensors for TB diagnosis.

In some countries with high TB burdens, access to fast, simple, cheap and reliable diagnostic strategies is one of the most important and urgent issues in controlling TB. The combination of nanotechnology and biosensing technology have indicated strong potentials to develop *Mtb* detection and for potential management in clinical diagnosis. In this review, we summarized a variety of nanobiosensors that are designed to detect different TB targets from nucleic acid to the whole bacteria. These methods offer great prospects in the development of rapid TB biosensing strategies with high sensitivity and accuracy. However, there are still some remaining shortcomings as following: 1) although the sensitivity and specificity seem to be satisfactory, most of those methods are lack of clinical detection data which could directly verify their clinical application prospect; 2) most of the fabrication procedures for those nanobiosensors are too complicated, which might affect the repeatability and applicability of these methods for clinical uses; 3) due to more attentions are needed to be paid in the development of inexpensive devices with high efficiency for the diagnosis of TB in latent phase; 4) most of those assays contain only one detection target which might result in false negative results in clinical detection.

Moreover, although lots of different nanobiosensors have been developed in recent decades for laboratorial analysis of TB associated samples, we can’t deny that the current progress of nanobiosensors is still far from the clinical application. The current clinical diagnostic methods, such as Ziehl-Neelsen (ZN) smear microscopy, Mycobacterial cultures, tuberculin skin testing (TST), interferon gamma release assays (IGRAs) and Xpert Mtb/RIF assay have been widely proved to be effective in TB diagnosis, which dramatically enhanced the ability of hospitals for the rapid treatment of TB, latent TB and drug-resistant TB. Some iconography techniques, such as X-ray and computerized tomography (CT), also significantly contribute to TB diagnosis by providing direct graphic evidences for active TB. Although some works have demonstrated the enhanced sensitivity of some novel nanobiosensors for clinical sample analysis beyond the current clinical strategy, their accuracy and sensitivity remain to be further confirmed in larger corhorts.

The current clinical diagnostic methods have developed very mature detection instruments that are suitable for clinical uses, however, the development of laboratorial instruments for nanobiosensors that are suitable for clinical uses also remain a critical issue for the future uses of nanobiosensors. Although some clinical detection strategies, such as IGRAs and Xpert Mtb/RIF assay, are restricted by the high cost issues for widely uses worldwide, the complicated materials and procedures in most reported nanobiosensing methods might also introduce the high cost issues for potential clinical uses of nanobiosensor in the future TB diagnosis. Thus, more attentions are still needed to be paid into the development of novel nanobiosensors with low cost and high sensitivity that are suitable for clinical uses.

We prospect that in the following decades, some simple and portable diagnostic strategies that are capable of multi-target detection will be evolved for TB diagnosis based on the nanobiosensing methods. With these expectations and hopes in mind, the final application of nanobiosensors from bench to clinical diagnosis would promote the pace on the road of global objectives for TB control.

## Data Availability

The original contributions presented in the study are included in the article/Supplementary Material, further inquiries can be directed to the corresponding authors.
